# Development of a clinical-dermoscopic model for the diagnosis of urticarial vasculitis

**DOI:** 10.1038/s41598-020-63146-w

**Published:** 2020-04-08

**Authors:** B. García-García, J. Aubán-Pariente, P. Munguía-Calzada, B. Vivanco, G. Argenziano, F. Vázquez-López

**Affiliations:** 1 Department of Dermatology, Central University Hospital of Asturias, Oviedo, Spain; 2 Department of Pathology, Central University Hospital of Asturias, Oviedo, Spain; 3 Dermatology Unit, University of Campania, Naples, Italy

**Keywords:** Skin diseases, Skin manifestations

## Abstract

The clinical criteria for the diagnosis of urticarial vasculitis lack accuracy, according to previous studies. The aim of the study was to assess the accuracy of a clinical and a clinical-dermoscopic model for the differential diagnosis of chronic spontaneous urticaria (CSU) and urticarial vasculitis (UV). Dermoscopic images of lesions with histopathologically confirmed diagnosis of CSU and UV were evaluated for the presence of selected criteria (purpuric patches/globules (PG) and red linear vessels). Clinical criteria of CSU and UV were also registered. Univariate and adjusted odds ratios were calculated. Multivariate regression analyses were conducted separately for clinical variables (clinical diagnostic model) and for both clinical and dermoscopic variables (clinical-dermoscopic diagnostic model). 108 patients with CSU and 27 patients with UV were included in the study. The clinical-dermoscopic model notably showed higher diagnostic sensitivity than the clinical approach (63% vs. 44%). Dermoscopic purpuric patches/globules (PG) was the variable that better discriminated UV, increasing by 19-fold the odds for this diagnosis. In conclusion, dermoscopy helps the clinical discrimination between CSU and UV. The visualization of dermoscopic PG may contribute to optimize decisions regarding biopsy in patients with urticarial rashes.

## Introduction

Urticarial vasculitis (UV) is a clinicopathologic entity characterized by urticarial lesions disclosing histopathologically leukocytoclastic vasculitis, mainly of postcapillary venules^1,2^. Recognizing UV is crucial given a possible association with systemic features. UV is classified into hypocomplementemic (HUV) and normocomplementemic (NUV). HUV is a rare systemic vasculitis, showing urticarial lesions and systemic manifestations, such as arthritis/arthralgia, glomerulonephritis, uveitis, recurrent abdominal pain or chronic obstructive pulmonary disease. More than half of patients have associated anti-C1q antibodies^2^. In contrast, NUV, is a skin-limited vasculitis^1,2^. UV may be associated to autoimmune connective tissue diseases, infections, drugs or neoplasia, although most of the cases are idiopathic^3,4^. UV is a rare condition, clinically overlapping with the more common chronic spontaneous urticaria (CSU). It is noteworthy that lesions of CSU and UV may be visually indistinguishable, and often represent a real diagnostic challenge for the clinician^5^.

A definitive diagnosis of UV requires a skin biopsy, which is performed when UV is suspected due to the presence of four classic clinical criteria: persistence of individual lesions, lasting more than 24 hours; presence of tenderness or a painful/burning sensation; purpura or dusky discoloration of the skin and resolution of lesions with residual hyperpigmentation^6^. The accuracy of this clinical approach has been questioned, as some studies have demonstrated absence of these features in a significant proportion of patients with UV^4–9^. Furthermore, even the need of a reassessment of diagnostic criteria of UV has been suggested^5^. The value of dermoscopy, a low-cost and rapid skin examination technique, for clinically discriminating common urticaria and UV has been proposed but in a few small-sized studies^10,11^. Dermoscopy of CSU typically reveal a red network of linear vessels, correlating with transient vasodilatation of horizontal subpapillary plexus^10,11^. In contrast, urticarial lesions of UV characteristically develop dermoscopic irregular/round small purpuric patches /globules (PG), derived from perivascular haemorrhage associated with inflammatory purpura^10,11^. We assessed and compare herein for the first time the accuracy of both a clinical and a clinical-dermoscopic model for discriminating urticarial vasculitis and chronic spontaneous urticaria.

The aim of this study was to investigate the value of dermoscopy for the differential diagnosis of urticariform rashes of CSU and UV, and to evaluate the accuracy of a clinical-dermoscopic diagnostic model versus a clinical approach for discriminating both diseases.

## Patients, Methods and Definitions

This was a retrospective, chart review, single center study developed from 2003 through 2014 at the department of Dermatology of a tertiary teaching university hospital (Central University Hospital of Asturias -HUCA-) in northern Spain. The study was approved by the Ethical Committee of Regional Clinical Research of the Principality of Asturias. All research was performed in accordance with relevant guidelines/regulations and informed consent was obtained from all participants. Inclusion criteria were a clinical diagnosis of an urticarial rash of at least 6 weeks of evolution (chronic spontaneous urticaria or urticarial vasculitis), complete information regarding clinical and dermoscopic features gathered during the medical interview and physical examination and a histopathologic confirmatory study. Exclusion criteria were: children, lack of patient´s consent, diagnosis of other erythematous-purpuric and persistent rashes (erythema multiforme, capillaritis, lymphocytic vasculitis, purpuric pityriasis rosea and insect bites) or absence of complementary data. Dermoscopic examination preceded biopsy, and both were performed on the same urticariform lesions. Since vasculitis is a dynamic process, and the most characteristic histological features develop in more recent lesions (neutrophils, haemorrhage, and leukocytoclasia)^21^, biopsies were taken from lesions less than 24 hours old. The lower leg location was excluded in order to avoid histological changes caused by venous stasis. Clinical criteria were registered on standardized questionnaires including: duration and persistence (more or less than 24 hours) of urticariform individual lesions; symptoms (pruritus, pain or burning sensation). Clinical lesions were described as wheals (transient); papules/ plaques (long lasting or undefined); erythema and purpuric areas were additionally discriminated by diascopy. Clinical variables assessed and selected for subsequent analysis in our study were the classical clinical signs of UV: persistence (urticariform lesions lasting more than 24 hours as referred by the patients), pain/burning sensation (as it was referred by the patients) and purpura/residual hyperpigmentation. Results of laboratory examinations were not evaluated given the clinical-dermoscopic nature of the study.

Lesions were examined with a 10× manual dermoscope (Delta 10; Heine Optotechnik, Herrsching, Germany, and later, Dermlite II Pro HR,3Gen Inc, CA, USA). Lesions were photographed with a digital dermoscopic camera (Dermaphot photographic equipment -Heine Optotechnik- and later Dermlite Foto System). A preliminary study was conducted in order to evaluate whether Delta 10 and Dermlite II Pro HR could give different dermoscopic observations in this setting, but no differences were found and consequently the dermoscopic observations were considered as a single data set. The dermoscopic procedure was applied taking into account that vessels recognition is the basis of dermoscopy of urticarial rashes^10–14^. Because vessels are visualized due to the red blood cells fulfilling and passing through them, dermoscopy was realized in a two-steps procedure: (a) non-contact dermoscopy (avoiding pressure), allowing recognition of both vascular and purpuric features; (b) dermoscopy applied over diascopy (applying a glass pressure over the lesion), blanching vessels meanwhile purpuric features persists.

Patients who both gave and signed an informed consent to perform a skin biopsy were recruited throughout the study period from a single outpatient dermatology office. Dermoscopic examination and photographs preceded biopsy. The retrospective scoring of dermoscopic structures, performed on dermoscopic images by two experienced dermatologists, included the following structures, according to previous studies:^10–14^ (1) **Dermoscopic vascular features**: (a) Round vessels (correlating with papillary vessels): dotted or globular, according to their size; b) Linear vessels (correlating with horizontal subpapillary vessels): simple, arboriform, and network structures, with a defined/blurred contour. (2) **Dermoscopic purpuric findings**: (a) Large homogeneous, structureless purpura (more frequently associated to non-inflammatory forms of dermal haemorrhage such as traumatic, senile or steroid purpura); b) Irregular/round small purpuric patches/globules (PG): perivascular haemorrhage, related to purpuric inflammatory processes (pigmented purpuric dermatoses, arthropods reactions, viral and drugs reactions, leukocytoclastic vasculitis, infective organisms). PG are blurred and appear first within a purpuric and later within an orange-brown background, which may obscure PG if it is prominent or when tissue necrosis appears. (c) Black/purpuric spots (subcorneal and subungual purpura); (**3) other dermoscopic structures**: haemorrhagic crusts; erosions/excoriations. In order to obtain a simple and feasible clinical-dermoscopic model for discriminating CSU and UV, and according to previous studies which include our previous experience^10–14^, we finally selected and scored only two dermoscopic features: purpuric patches/globules (PG) and red linear vessels.

Histological criteria for diagnosis of UV were: perivascular and interstitial neutrophilic infiltrate; signs of karyorrhexis (nuclear dust); extravasated erythrocytes and deposition of fibrin in the vessels. Histological criteria for diagnosis of CSU were superficial and deep perivascular and interstitial infiltrate of lymphocytes, neutrophils and eosinophils devoid of karyorrhexis and vascular fibrin deposition.

### Statistical analysis

Statistical analysis was performed using SPSS software (IBM Corp. Release 2013. IBM SPSS Statistics for Windows, version 21.0. Armonk, NY: IBM Corp). Univariate and multivariate analyses were conducted by logistic regression. Crude odds ratio (OR), adjusted OR and the corresponding 95% confidence interval (CI) and p-value were obtained for every clinical and dermoscopic variable. Multivariate analysis was conducted separately for clinical variables and subsequently for both clinical and dermoscopic variables altogether. Goodness of fit of the multivariate analyses was examined by adjusted R^2^, likelihood ratio test and correct classification percentage. Specificity, sensitivity, positive predictive value (PPV) and negative predictive value (NPV) were extracted from classification tables for both diagnostic models (the clinical and the clinical-dermoscopic) according to standard formulas. We considered statistically significant a 2-sided p-value of 0.05.

## Results

In all, 135 patients with urticariform rashes and fulfilling all inclusion criteria were finally recruited (84 female, 51 male; mean age 50 years; range 20–79 years). Of those, 108 (80%) patients had CSU and 27 (20%) had UV. Descriptive results of clinical signs, symptoms and dermoscopic features for both histological groups are quoted in Table 1.

**Table 1:** Frequencies and univariate analysis of the five clinical and dermoscopic variables assessed in our chronic spontaneous urticaria and urticarial vasculitis patients.

Predictor	Chronic spontaneous urticaria (n = 108) n (%)	Urticarial vasculitis (n = 27) n (%)	p-value	OR	95% CI
Persistence (wheals ˃24 hours)	28 (25.9)	19 (70.4)	**<0.001**	**6.79**	**2.67**–**17.22**
Pain/Burning	17 (15.7)	7 (25.9)	0.216	1.87	0.69–5.12
Purpura/residual hyperpigmentation	10 (9.3)	13 (48.1)	**<0.001**	**9.10**	**3.36**–**24.65**
DC red linear vessels	92 (85.2)	20 (74.1)	0.17	0.50	0.18–1.37
DC purpuric patches/globules (PG)	11 (10.2)	19 (70.4)	**<0.001**	**20.94**	**7.44**–**58.96**

Statistically significant values are shown in bold. DC: dermoscopic.

**Figure 1:**
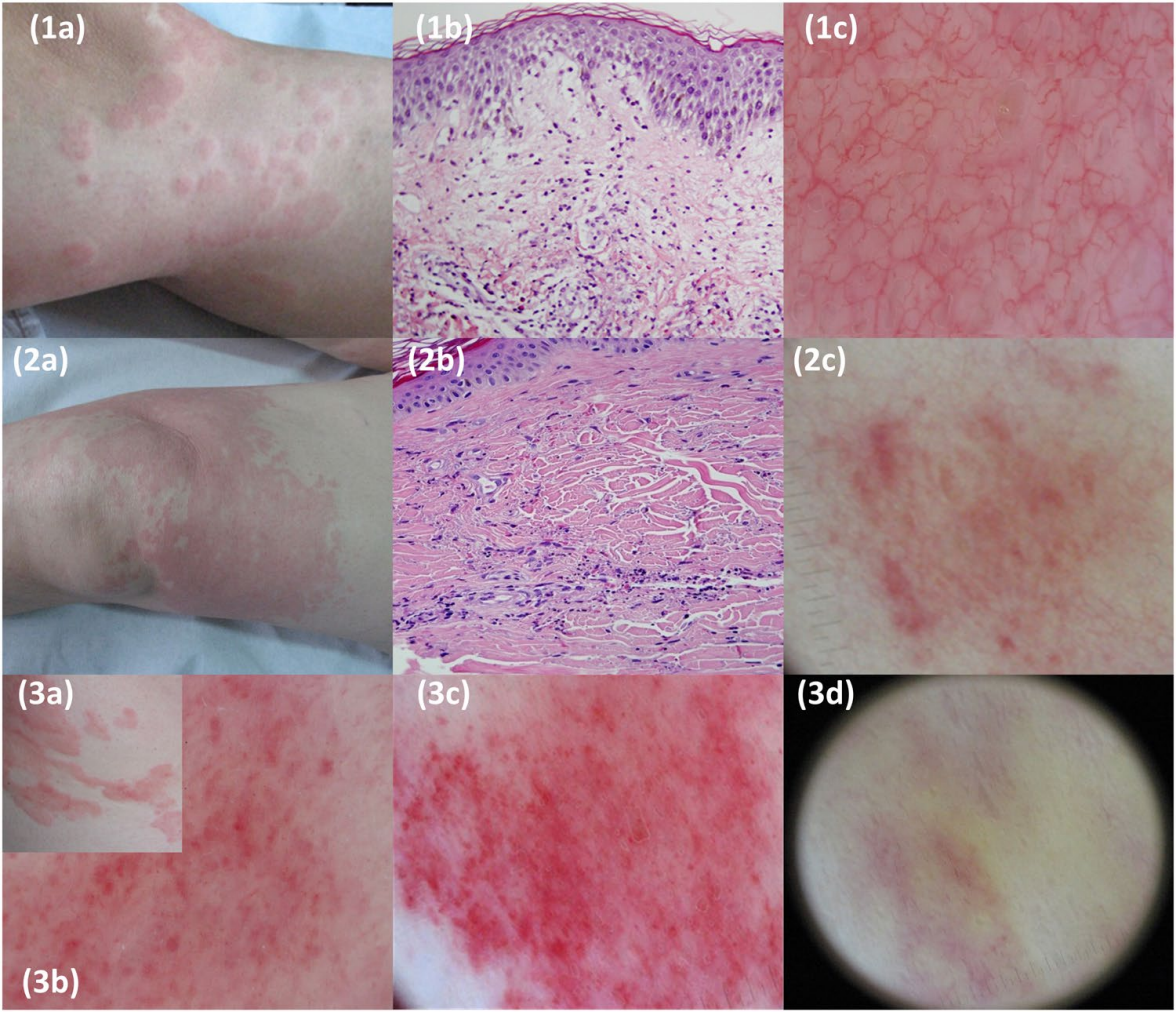
Clinical, histological and dermoscopic features of chronic spontaneous urticaria (patient 1) and urticarial vasculitis (patients 2 and 3). Patient 1. 1 A: Multiple wheals on the legs of a woman with an urticarial rash. 1B: edema in papillary and upper reticular dermis and a mixed inflammatory infiltrate constituted by lymphocytes, eosinophils and occasional neutrophils (H&E, original magnification ×20). 1 C: dermoscopic well-defined network of red lines. Purpuric patches are absent. Patient 2. 2 A: persistent isolated and confluent erythemato-edematous urticarial papules and plaques located on a leg. 2B: A predominantly neutrophilic inflammatory infiltrate affecting the vascular walls and associated with blood extravasation and nuclear dust (karyorrhexis) (H&E, original magnification x40). 2 C: dermoscopic small irregular purpuric patches and red lines. Patient 3. 3 A: urticarial erythematous papules and plaques located on the trunk. 3B, 3 C: different degrees of dermoscopic blurred small purpuric patches and red lines on an erythematous background. 3D: dermoscopy of a residual, long lasting lesion disclosing a yellow/orange residual discoloration.

As regards dermoscopic findings, red linear vessels were observed in the majority of both CSU and UV patients (85% and 74% respectively) but PG were highly discriminative, being present in most of the patients with UV (n = 19, 70.4%) but only in a minority of patients with CSU (n = 11; 10.2%) (Fig. 1). Univariate analysis yielded statistical significance for two clinical variables (persistence of lesions, and purpura/residual hyperpigmentation) and one dermoscopic feature (PG), increasing the likelihood for UV by 7-fold, 9-fold and 21-fold respectively (Table 1).

A multivariate regression analysis of a clinical model (where only clinical variables were entered), maintained the significance for persistence (OR: 4.97; 95% CI 1.85–13.35) and purpura/residual hyperpigmentation (OR 6.34; 95% CI 2.19–18.36) (Table 2). Finally, the multivariate regression analysis of a clinical-dermoscopic model (based on both clinical and dermoscopic variables), showed that dermoscopic PG were an independent positive predictor for UV. Only one clinical variable -persistence of lesions- maintained significance. This final model revealed a 19-fold increase in the odds for UV when dermoscopic PG were present and a 6-fold increase for persistence of lesions, when the diagnosis of UV was compared with CSU (Table 2). Goodness of fit was examined by correct classification percentage (88.9%), adjusted R^2^ (0.503) and a significant likelihood ratio test (p < 0.05). Interestingly, it was remarkable that sensitivity was notably higher for the clinical-dermoscopic approach than for the clinical model (63% vs. 44%) (Table 3).

**Table 2:** Diagnostic models (clinical and clinical-dermoscopic) for differential diagnosis of chronic spontaneous urticaria and urticarial vasculitis.

Diagnostic model	Predictor	p-value	OR	95% CI
Clinical model	Persistence (wheals ˃24 hours)	<0.001	4.97	1.85–13.35
	Purpura/residual hyperpigmentation	<0.001	6.34	2.19–18.36
Clinical-dermoscopic model	DC purpuric patches/globules (PG)	<0.001	19.42	5.79–65.15
	DC red linear vessels	0.011	0.13	0.03–0.63
	Persistence (wheals ˃24 hours)	0.006	5.65	1.64–19.44

Multivariate analysis with only clinical variables entered (clinical model) and all variables (clinical-dermoscopic model): adjusted clinical and clinical-dermoscopic predictors for urticaria vasculitis from 135 patients.

**Table 3:** Sensitivity, Specificity, Positive Predictive Value (PPV) and Negative Predictive Value (NPV) of both diagnostic models, obtained by multivariate logistic regression analysis.

Diagnostic models	Sensitivity (%)	Specificity (%)	PPV (%)	NPV (%)	Correct diagnosis (%)
Clinical	44.4	97.2	80	87.5	86.7
Clinical-dermoscopic	63	95.4	77.3	91.2	88.9

## Discussion

Dermoscopy is a non-invasive skin magnification technique, which allows a real *in vivo* subclinical exploration of the skin. Dermoscopy improves and completes clinical examination by revealing morphologic structures scarcely visible or invisible on the standard naked-eye physical examination, enhancing the most basic of diagnostic functions in dermatology: the visual inspection^15^. Dermoscopy has a well demonstrated value in the diagnosis of skin tumours, especially melanoma, but also of many non-tumoral dermatosis, such as infections and infestations, hair or nail diseases and inflammatory skin conditions^15–17^. In addition, nail fold dermoscopy may be applied for the screening of suspected connective tissue diseases instead of classic capillaroscopy^18^. In fact, this valuable low-cost device has even been considered to have in dermatology a role similar to the stethoscope of general practitioners^19^.

The value of dermoscopy for the differential diagnosis between common urticaria and urticarial vasculitis has been proposed but in small series^10,11^. In the present study, we confirm this in a larger series of patients, adding for the first time the evaluation of a clinical-dermoscopic model versus a clinical model for differentiating CSU and UV. It is our premise that dermoscopic findings should always be interpreted within the overall clinical context of the patient, and integrated with history and standard clinical examination. Consequently, we developed a clinical-dermoscopic diagnostic model instead of a pure dermoscopic model for differentiating both diseases. Our observations mainly revealed that sensitivity for differentiating UV from CSU is improved when a clinical-dermoscopic model is applied in comparison with a clinical model (63% vs 44%).

The application of a clinical model for differentiating common urticaria and UV is the gold standard at the present time. The classical clinical signs (urticariform lesions lasting more than 24 hours, pain/ burning sensation, purpura/residual hyperpigmentation) help to suspect UV, and establish whether a biopsy is needed (to confirm a UV diagnosis) or not (in case of common urticaria). Nevertheless, there are studies reporting a lack of efficacy of this clinical model, taking into account the variable frequencies of these clinical signs (Table 4)^4–9^. In a study including 47 patients with biopsy proven UV, Tosoni *et al*. found that most of them did not show the classic clinical features of UV: only 57% of patients referred lesions lasting more than 24 hours and pain was reported in only 8,6%. Consequently, they suggested the need of a reassessment of the diagnostic criteria of UV^5^. Additional strategies have included outlining the contour of the lesions for evaluating the eventual persistence of individual lesions and performing a biopsy according to the response to the treatment with oral antihistamines instead of considering the clinical features^4,5^. These methods imply the cons of being time and cost-consuming and invasive, in case of performing routine biopsies. Histopathologically, the essential criteria for the diagnosis of UV is the presence of karyorrhexis (nuclear dust), joined by extravasated erythrocytes and, at time, by fibrin deposits within walls of vessels^6,20,21^. Vascular fibrinoid necrosis is considered rare or absent in UV. Biopsy timing (optimally within the first 24–48 hours) is important to achieve a characteristic picture of UV^20,21^.

**Table 4:** Clinical criteria of urticarial vasculitis (proportion of patients) according to literature and our study.

Literature: Author, year of publication and reference.	UV patients N	Persistence (wheals ˃24 hours) N (%)	Pain/burning N (%)	Purpura/ residual hyperpigmentation N (%)
Mehregan *et al*.^6^	72	46 (63.8)	23 (31.9)	25 (34.7)
Lee *et al*.^8^	22	22 (100)	10 (45.5) (pain) 15 (68.2) (burning)	18 (81.8)
Dincy *et al*.^4^	68	61 (89.7)	22 (32.3)	17 (25)
Tosoni *et al*.^5^	47	20 (42.6)	4 (8.6) (pain) 6 (12.8) (burning)	3 (6.4)
Kulthanan *et al*.^7^	64	60 (93.8)	28 (43.8) (pain) 12 (18.8) (burning)	53 (82.8)
Moreno *et al*.^9^	15	14 (93.3)	2 (13.3)	9 (60)
Garcia *et al*. (present study)	27	19 (70.4)	7 (25.9)	13 (48.1)

UV: urticarial vasculitis.

Our observations showed that lesions persistence and purpura/residual hyperpigmentation were the most frequent clinical variables in UV patients, in line with previous investigations (Table 4). Both variables yielded statistical significance, increasing the likelihood for UV by 7-fold and 9-fold respectively. In contrast, pain/burning sensation was only referred by 26% of patients, and did not reach significance. Considering only these clinical variables, the global accuracy of the clinical diagnostic model for discriminating UV and CSU was 87%, with 97% specificity for CSU, but with only a low 44% sensitivity for UV. Interestingly, when dermoscopy was added to the patient evaluation, the clinical-dermoscopic model increased sensitivity to 63%, while maintaining accuracy (89%), thus outlining the role of dermoscopy in improving the correct non-invasive diagnosis of UV. This improvement was mainly achieved by the detection of subclinical purpuric patches/globules (PG) that became visible by the use of dermoscopy. Taking into account that the degree of dermal haemorrhage in UV lesions is variable, ranging from evident to unapparent purpura, identification of dermoscopic PG may be especially useful for those UV lesions with minimal clinical purpuric areas. Indeed, the presence of dermoscopic PG was the most valuable criteria for discriminating CSU and UV by multivariate analysis (19-fold increase in the odds for UV), while clinical persistence of lesions increased it by 6-fold. Although we did not investigate this topic, it is also of interest that that PG might be present even in early UV lesions^11^.

Under dermoscopy, the wheals of CU mainly revealed red linear vessels, which correlate with ectatic, horizontally oriented, subpapillary vessels, and reflect a process of transient vasodilatation of dermal capillaries. They were obscured when prominent oedema was present (negative areas). Purpuric structures or patches were rare in CSU and must be differentiated from erosions/crusts and from red round vessels (vertical papillary vessels) which appear as red dots with a clear contour, located along linear vessels, and disappearing after diascopy^14^. Urticarial lesions of UV also revealed linear vessels but, in contrast to CSU, they frequently showed blurred irregular/round purpuric structures (PG) within a purpuric (early stage) or orange-brown (late stage) background. Limitations of our study include the retrospective design and the number of patients evaluated.

In conclusion, dermoscopy helps the clinical discrimination between chronic spontaneous urticaria and urticarial vasculitis by improving the sensitivity of the standard clinical examination (visual inspection). Dermoscopy enhances visualization of subclinical purpuric patches, which are highly indicative of an underlying vasculitis when confronted with common urticaria. This technique may contribute to optimize decisions regarding biopsy in patients with urticarial rashes, so commonly attended in daily clinical practice.
